# Recursive partitioning staging system based on the log odds of the negative lymph node/T stage ratio in colon mucinous adenocarcinoma

**DOI:** 10.3389/fimmu.2024.1472620

**Published:** 2024-12-20

**Authors:** Huajun Cai, Jintao Zeng, Ye Wang, Jinfu Zhuang, Xing Liu, Guoxian Guan

**Affiliations:** Department of Colorectal Surgery, National Regional Medical Center, Binhai Campus of the First Affiliated Hospital, Fujian Medical University, Fuzhou, China

**Keywords:** colon mucinous adenocarcinoma, LONT, prognosis, recursive partitioning analysis, staging system

## Abstract

**Background:**

This study aimed to investigate the prognostic significance of the log odds of negative lymph nodes/T stage ratio (LONT) and develop an efficient prognostic staging system using LONT in patients with colon mucinous adenocarcinoma (MAC).

**Methods:**

This study included 5,236 patients diagnosed with colon MAC obtained from the Surveillance, Epidemiology, and End Results database. The Kaplan–Meier method, subgroup analysis, receiver operating characteristic (ROC) curve, and Cox proportional hazard regression model were used to determine the clinical outcomes. Recursive partitioning analysis (RPA) was used to develop a novel prognostic system.

**Results:**

The 1-, 3-, and 5-year ROC curves, used to predict cancer-specific survival (CSS) and overall survival (OS), demonstrated that the areas under the ROC curve for LONT were superior to those of pT, pN, and pTNM stages. Additionally, a lower LONT was correlated with worse clinical outcomes. The LONT classification efficiently differentiated the prognosis of patients in terms of OS and CSS. Multivariate Cox analyses revealed that LONT was an independent prognostic factor for both CSS and OS. Based on the pT stage and LONT, a novel prognostic staging system was developed using RPA, demonstrating a good prognostic predictive performance.

**Conclusion:**

A lower LONT was associated with worse survival in patients with colon MAC. The pT stage and LONT-based prognostic staging system facilitated risk stratification in these patients.

## Introduction

Colorectal cancer ranks third globally in terms of incidence and second in mortality rates (1, 2). Adenocarcinoma is the predominant pathological type of colon cancer. Mucinous adenocarcinoma (MAC) constitutes approximately 5%–15% of colon cancer cases and often demonstrates advanced staging and lymph node metastasis (3, 4). However, the prognosis of colon MAC remains debatable (5).

The American Joint Committee on Cancer (AJCC) tumor-node-metastasis (TNM) staging system, comprising pT and pN stages, is widely used for staging patients with colon MAC. However, the pN stage exclusively considers the number of positive lymph nodes (PLNs), neglecting the significance of cleared negative lymph nodes (6–8). Considering the correlation between lymph node clearance and cancer prognosis, the lymph node ratio (LNR) and log odds of positive lymph nodes (LODDS) have been identified as prognostic indicators. LNR is defined as the ratio of PLNs to the total number of regional lymph nodes. However, this formula indicates that its prognostic utility is limited in patients with negative lymph nodes. Conversely, the LODDS accounts for both PLN and the number of negative lymph nodes, providing a comprehensive evaluation. Despite their significance, these metrics were developed solely on the basis of lymph node status and count without adjustment for clinical risk factors.

The log odds of negative lymph nodes/T stage ratio (LONT) combines data from the pT stage and negative lymph nodes. It adjusts lymph node–related information by incorporating T staging. Xie et al. (9) pioneered the application of LONT in developing a nomogram for gastric cancer. Their study revealed that patients with different TNM stages but the same LONT exhibited similar risk levels, indicating the superior prognostic capability of their model compared to TNM staging alone. Moreover, LONT has been used to develop nomograms for thyroid cancer without metastases (10) and bladder cancer (11), demonstrating better predictive capabilities than the TNM staging system. However, the use of LONT in colon MAC remains unexplored.

In this study, a population-based cohort from the Surveillance, Epidemiology, and End Results (SEER) database was used to investigate the association between LONT and the survival of patients with colon MAC. We developed a prognostic staging system on the basis of LONT to predict the survival of patients with colon MAC using recursive partitioning analysis (RPA).

## Methods

### Cohort characteristics

The cohort included patients with colon MAC identified from the SEER database between 2006 and 2015 using SEER*Stat software. The inclusion criteria were as follows: (1) diagnosis of colon MAC as per the International Classification of Diseases of Oncology (ICD-O-3), (2) radical surgical treatment including lymph node dissection, and (3) the absence of distant metastasis. The exclusion criteria included the following: (1) age > 75 years at diagnosis, (2) diagnosis or surgical identification of distant metastases, (3) other concurrent primary tumors, and (4) incomplete clinicopathological or follow-up data. The SEER cohort included 5,236 patients. This study was approved by the hospital ethics committee.

### Data extraction

The patient data collected included age at diagnosis, sex, race, tumor size, primary tumor site, AJCC TNM stage, number of examined lymph nodes, counts of positive and negative lymph nodes, cancer-specific survival (CSS), overall survival (OS) status, and follow-up duration. The pN staging was adjusted on the basis of the latest AJCC TNM staging system.

### LONT classification

The log odds of (negative lymph nodes + 1)/T stage ratio were calculated as LONT. The number of negative lymph nodes was equal to the number of lymph nodes examined minus the number of PLNs, and pT1, pT2, pT3, and pT4 were assigned values of 1, 2, 3, and 4, respectively. LONT was then grouped at intervals of 0.2. Kaplan–Meier analysis was conducted on the adjacent groups, and those exhibiting similar CSS were combined into a common subgroup.

### Statistical analysis

Statistical analyses were performed using R software (version 3.6.3) and Statistical Package for the Social Sciences software (version 22.0). Receiver operating characteristic (ROC) curve analysis was conducted to assess the prognostic predictive value of the pT stage, pN stage, pTNM stage, and LONT classification. Disparities within subgroups stratified by distinct clinicopathological characteristics were assessed using Kaplan–Meier analysis. The independent risk factors for CSS and OS were identified using univariate and multivariate Cox regression analyses. A novel tumor prognostic staging system was reclassified using RPA, which is accessible online (http://rpa.renlab.org) (12). A *P* < 0.05 indicated a statistically significant difference.

## Results

### Baseline characteristics

The SEER cohort included 5,236 patients, and their clinical characteristics are summarized in **Table 1**. The mean age of the patients was 60.1 ± 11.2 years, and 45.6% were women. The number of patients with pT3 and pT4 tumors was 3,333 (63.7%) and 1,123 (21.4%), respectively. PLNs were detected in 2,274 patients (43.4%).

**Table 1:** The clinicopathologic features of SEER cohort.

Characteristics	SEER Cohort
	N (%)
Age, years	60.1 ± 11.2
Sex	
Female	2,439 (45.6)
Male	2,797 (53.4)
Race	
White	4,113 (78.6)
Black	714 (13.6)
Other/Unknown	409 (7.8)
Tumor size, cm	6.0 ± 3.8
Tumor site	
Proximal colon	3,851 (73.5)
Distal colon	1,385 (26.5)
pT stage	
T1	180 (3.4)
T2	600 (11.5)
T3	3,333 (63.7)
T4	1,123 (21.4)
pN stage	
N0	2,962 (56.6)
N1a	644 (12.3)
N1b	656 (12.5)
N2a	468 (8.9)
N2b	506 (9.7)
pTNM stage	
Stage I	619 (11.8)
Stage II	2,343 (44.7)
Stage III	2,274 (43.4)

SEER, Surveillance, Epidemiology, and End Results; TNM, tumor-node-metastasis.

### The prognostic value of LONT

**Figure 1:**
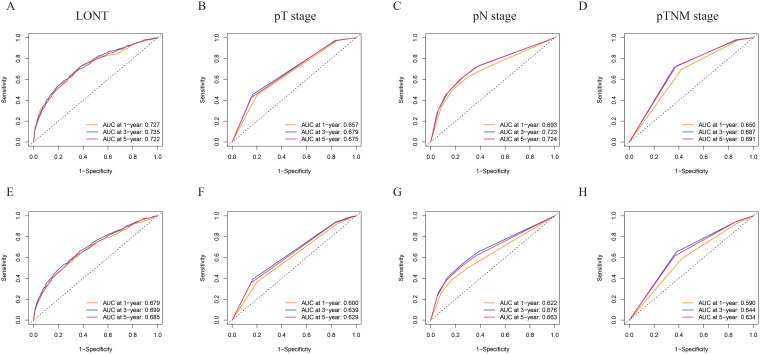
ROC curves of 1-, 3-, and 5-year CSS and OS prediction of **(A, E)** LONT; **(B, F)** pT stage; **(C, G)** pN stage; **(D, H)** pTNM stage. LONT demonstrated superior predictive performance compared to the pT stage, pN stage, and pTNM stage (AUC in the bottom right of the figure). ROC, receiver operating characteristic curve; CSS, cancer-specific survival; OS, overall survival; LONT, the log odds of negative lymph nodes/T stage ratio; SEER, Surveillance, Epidemiology, and End Results; AUC, area under the ROC curve.

To investigate the prognostic assessment capabilities of LONT, ROC analysis was used to compare the predictive abilities of LONT, pT, pN, and pTNM stages for 1-, 3-, and 5-year survival LONT demonstrated superior predictive performance compared to those of the pT, pN, and pTNM stages (**Figure 1**). The area under the ROC curve (AUC) values for LONT in predicting 1-, 3-, and 5-year CSS were 0.727, 0.735, and 0.727, respectively (**Figures 1A–D**). Additionally, the AUC values for predicting 1-, 3-, and 5-year OS were 0.679, 0.699, and 0.685, respectively (**Figures 1E–H**). These results indicated that LONT is a valuable prognostic factor that warrants further investigation.

### Prognostic factors of CSS and OS

Cox regression analysis was further refined to identify independent prognostic factors in patients with colon MAC. Univariate Cox analysis revealed that age, tumor size, tumor site, pTNM stage, and LONT were significantly associated with CSS (*P* < 0.05). Multivariate Cox analysis demonstrated that age (*P* < 0.001), tumor size (*P* < 0.001), pTNM stage (*P* < 0.001), and LONT (*P* < 0.001) were independently associated with CSS (**Table 2**). The results of Cox regression analysis for identifying independent prognostic factors for OS are presented in **Table 3**. Univariate Cox analysis revealed that age, sex, tumor size, pTNM stage, and LONT were significantly associated with OS (*P* < 0.05). Multivariate Cox analysis confirmed that age (*P* < 0.001), sex (*P* < 0.001), tumor size (*P* = 0.004), pTNM stage (*P* < 0.001), and LONT (*P* < 0.001) were independently associated with OS. These findings underscore that LONT is an independent risk factor for both CSS and OS in patients with colon MAC.

**Table 2:** Univariate and multivariable Cox analyses of CSS for patients with colon MAC.

Characteristics	Univariate			Multivariate		
	HR	95% CI	*P*-value	HR	95% CI	*P*-value
Age, years	1.009	1.003–1.015	0.004	1.015	1.009–1.021	<0.001
Sex						
Female	Ref.					
Male	1.124	0.990–1.278	0.072			
Race			0.177			
White	Ref.					
Black	1.182	0.991–1.410	0.063			
Other/unknown	1.034	0.812–1.317	0.786			
Tumor size, cm						
≤6.0	Ref.			ref.		
>6.0	1.307	1.151–1.485	<0.001	1.306	1.148–1.485	<0.001
Tumor site						
Proximal colon	Ref.			ref.		
Distal colon	1.162	1.011–1.337	0.035	0.988	0.857–1.140	0.872
pTNM stage			<0.001			<0.001
Stage I	Ref.			ref.		
Stage II	3.478	2.207–5.480	<0.001	2.510	1.589–3.965	<0.001
Stage III	10.763	6.899–16.792	<0.001	6.372	4.062–9.994	<0.001
LONT	0.118	0.101–0.139	<0.001	0.177	0.149–0.211	<0.001

CSS, cancer-specific survival; MAC, mucinous adenocarcinoma; HR, hazard ratio; TNM, tumor-node-metastasis; LONT, the log odds of negative lymph nodes/T stage ratio.

**Table 3:** Univariate and multivariable Cox analyses of OS for patients with colon MAC.

Characteristics	Univariate			Multivariate		
	HR	95% CI	*P-*value	HR	95% CI	*P*-value
Age, years	1.025	1.020–1.031	<0.001	1.029	1.023–1.035	<0.001
Sex						
Female	Ref.			ref.		
Male	1.190	1.069–1.324	0.001	1.236	1.110–1.376	<0.001
Race			0.229			
White	Ref.					
Black	1.076	0.925–1.251	0.342			
Other/unknown	0.865	0.697–1.073	0.188			
Tumor size, cm						
≤6.0	Ref.			Ref.		
>6.0	1.120	1.006–1.248	0.039	1.177	1.055–1.313	0.004
Tumor site						
Proximal colon	Ref.					
Distal colon	1.116	0.992–1.255	0.067			
pTNM stage			<0.001			<0.001
Stage I	Ref.			Ref.		
Stage II	1.394	1.113–1.747	0.004	1.077	0.856–1.354	0.526
Stage III	2.916	2.345–3.625	<0.001	1.981	1.581–2.438	<0.001
LONT	0.169	0.146–0.196	<0.001	0.217	0.186–0.254	<0.001

OS, overall survival; MAC, mucinous adenocarcinoma; HR, hazard ratio; TNM, tumor-node-metastasis; LONT, the log odds of negative lymph nodes/T stage ratio.

### LONT classification

**Figure 2:**
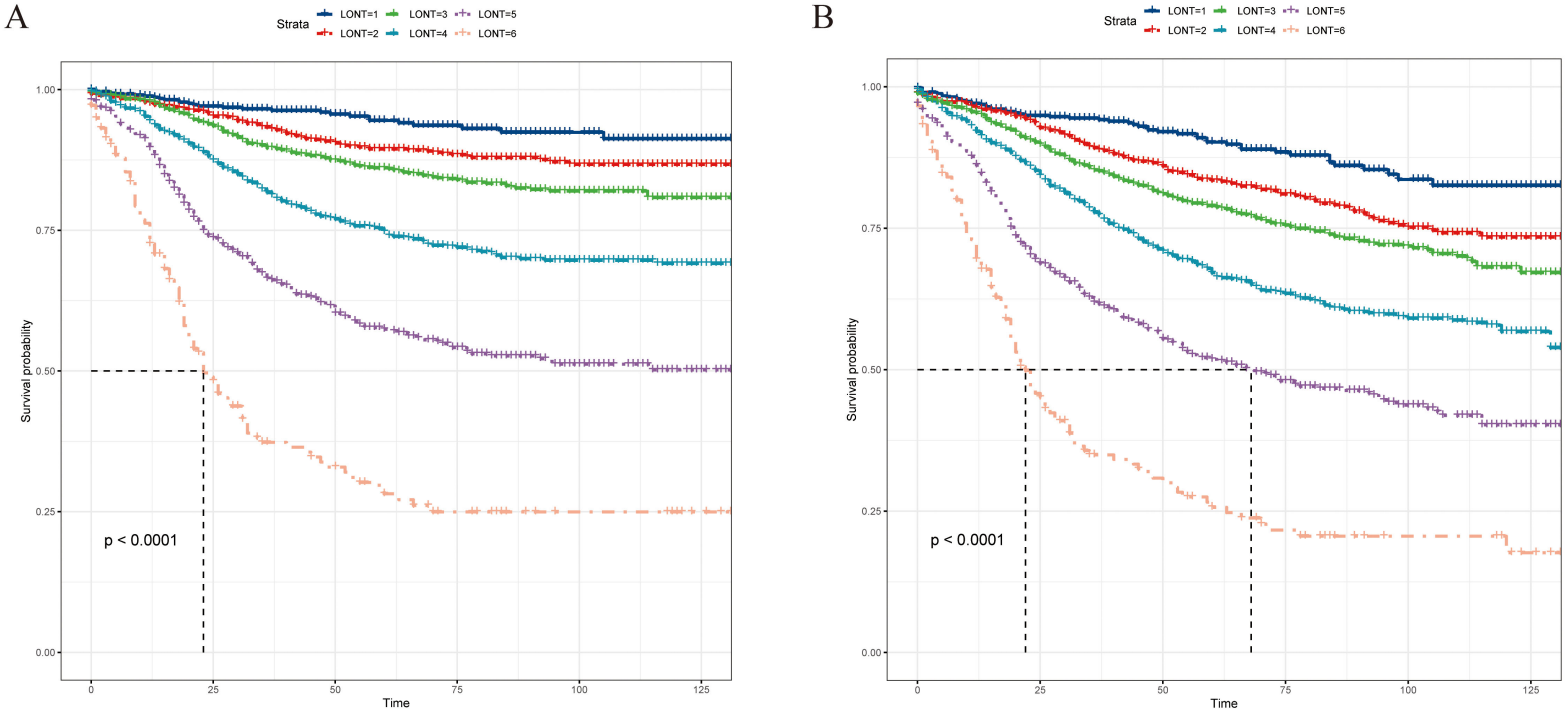
Kaplan–Meier curves for **(A)** CSS and **(B)** OS according to the LONT classification, and survival differences were observed (*P* < 0.05). CSS, cancer-specific survival; OS, overall survival; LONT, the log odds of negative lymph nodes/T stage ratio; SEER, Surveillance, Epidemiology, and End Results.

We divided LONT into six subgroups on the basis of similar CSS (**Table 4**): LONT1 (1.1 < LONT ≤ 1.7), LONT2 (0.9 < LONT ≤ 1.1), LONT3 (0.7 < LONT ≤ 0.9), LONT4 (0.5 < LONT ≤ 0.7), LONT5 (0.1 < LONT ≤ 0.5), and LONT6 (−0.7 < LONT ≤ 0.1). The 5-year CSS rates in these subgroups were 94.5%, 89.4%, 86.1%, 74.8%, 57.4%, and 28.2%, respectively. There were significant differences in the 5-year CSS and OS rates between the subgroups (*P* < 0.05; **Figure 2**).

**Table 4:** Five-year CSS in LONT subgroups.

LONT	N	5-year CSS (%)	*P*-value*	LONT classification	N	5-year CSS (%)	*P*-value*
−0.7 < LONT ≤ −0.5	13	19.6	0.945	−0.7 < LONT ≤ 0.1	177	28.2	<0.001
−0.5 < LONT ≤ −0.3	32	29.3	0.719				
−0.3 < LONT ≤ −0.1	43	20.8	0.150				
−0.1 < LONT ≤ 0.1	89	32.8	0.018				
0.1 < LONT ≤ 0.3	120	50.5	0.058	0.1 < LONT ≤ 0.5	538	57.4	<0.001
0.3 < LONT ≤ 0.5	418	59.3	<0.001				
0.5 < LONT ≤ 0.7	1,284	74.8	<0.001	0.5 < LONT ≤ 0.7	1284	74.8	<0.001
0.7 < LONT ≤ 0.9	1,592	86.1	0.004	0.7 < LONT ≤ 0.9	1592	86.1	0.040
0.9 < LONT ≤ 1.1	1,136	89.4	0.020	0.9 < LONT ≤ 1.1	1136	89.4	0.007
1.1 < LONT ≤ 1.3	399	94.8	0.950	1.1 < LONT ≤ 1.7	509	94.5	—
1.3 < LONT ≤ 1.5	99	92.3	0.415				
1.5 < LONT ≤ 1.7	11	100.0	—				

CSS, cancer-specific survival; LONT, the log odds of negative lymph nodes/T stage ratio.

*Comparison between adjacent subgroup groups.

### The subgroups analysis of the LONT classification

**Figure 3:**
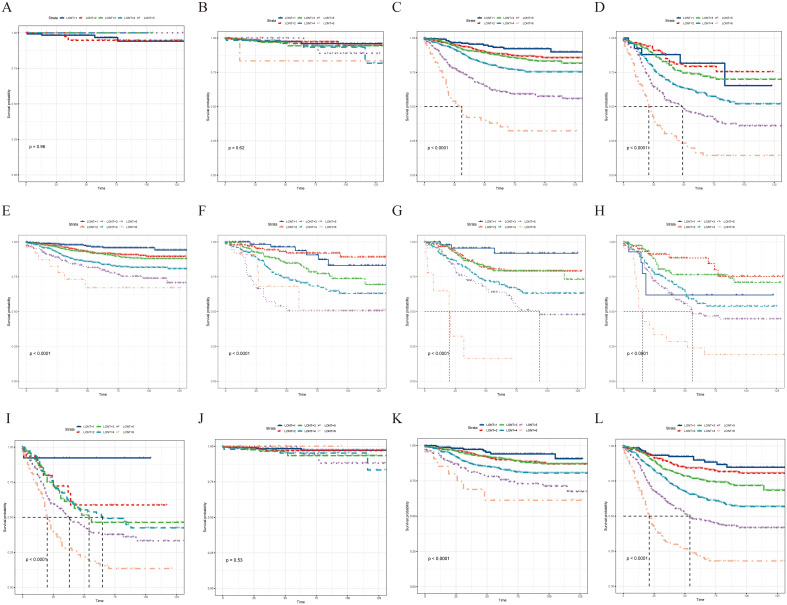
LONT differentiated the presentation of CSS in subgroup analyses. Kaplan–Meier curves evaluating CSS according to LONT classification in **(A–D)** pT stages I–III, **(E–I)** pN stages N0–N2b, and **(J–L)** pTNM stages I–III. CSS, cancer-specific survival; LONT, the log odds of negative lymph nodes/T stage ratio; TNM, tumor-node-metastasis.

**Figure 4:**
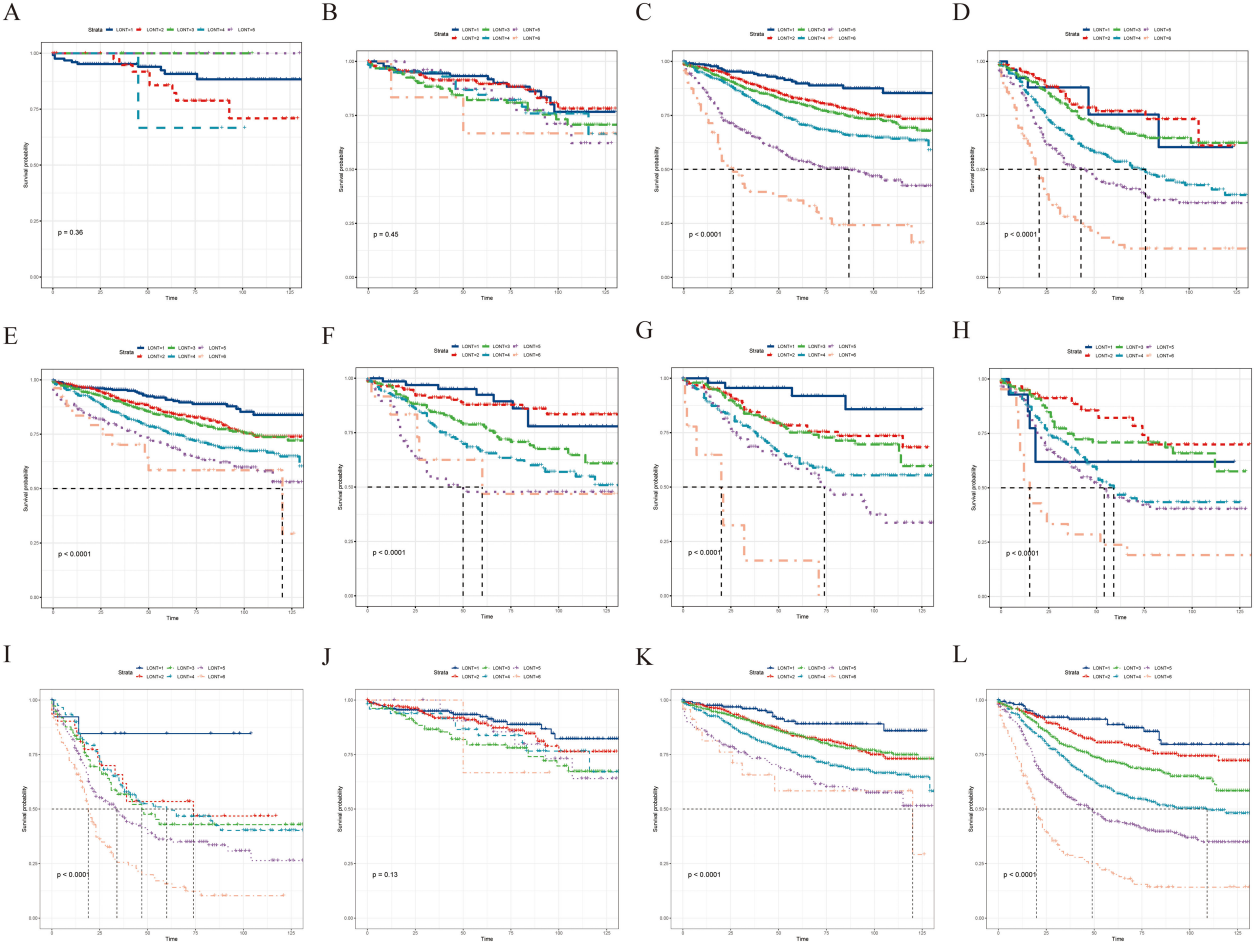
LONT differentiated the presentation of OS in the subgroup analyses. Kaplan–Meier curves evaluating OS according to LONT classification in **(A–D)** pT stages I–III, **(E–I)** pN stages N0–N2b, and **(J–L)** pTNM stages I–III. OS, overall survival; LONT, the log odds of negative lymph nodes/T stage ratio; TNM, tumor-node-metastasis.

Subgroup analysis revealed that the LONT classification could identify CSS differences in pT3, pT4, pN0, pN1a, pN1b, pN2a, pN2b, pTNM II, and pTNM III stages (*P* < 0.05; **Figure 3**). However, non-significant differences were observed in the pT1 (*P* = 0.96), pT2 (*P* = 0.62), and pTNM I stage (*P* = 0.53) subgroups. The same trend was observed during the OS evaluation (**Figure 4**). Specifically, the LONT classification could distinguish OS differences in pT3 stage, pT4 stage, pN0 stage, pN1a stage, pN1b stage, pN2a stage, pN2b stage, pTNM II stage, and pTNM III stage (*P* < 0.05). However, non-significant differences were observed in the pT1 (*P* = 0.36), pT2 (*P* = 0.45), and pTNM I (*P* = 0.13) subgroups.

### Development of a novel prognostic staging system

**Figure 5:**
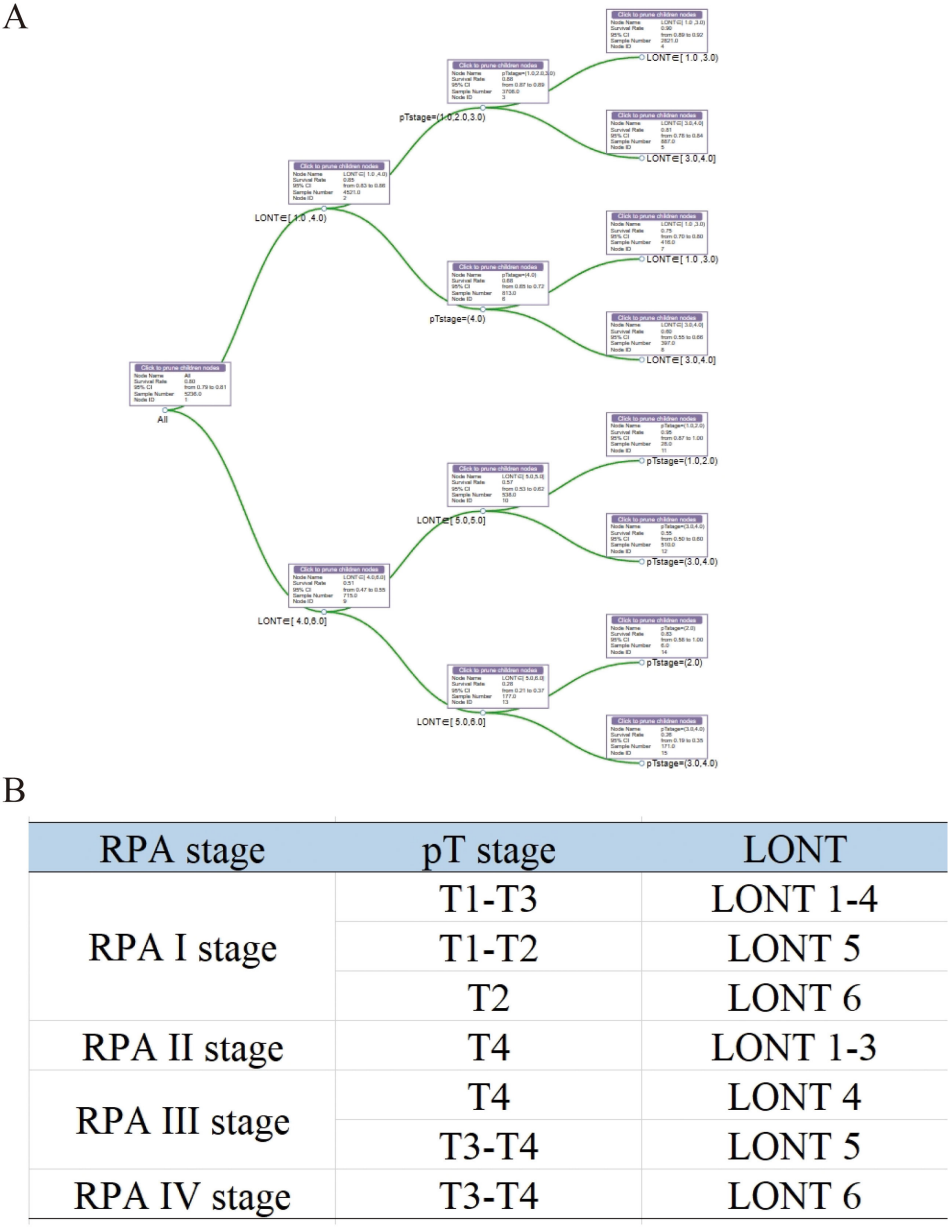
Development of an RPA staging system for prognostic stratification. **(A)** RPA for patients with colon MAC. **(B)** The RPA staging system. MAC, mucinous adenocarcinoma.

**Figure 6:**
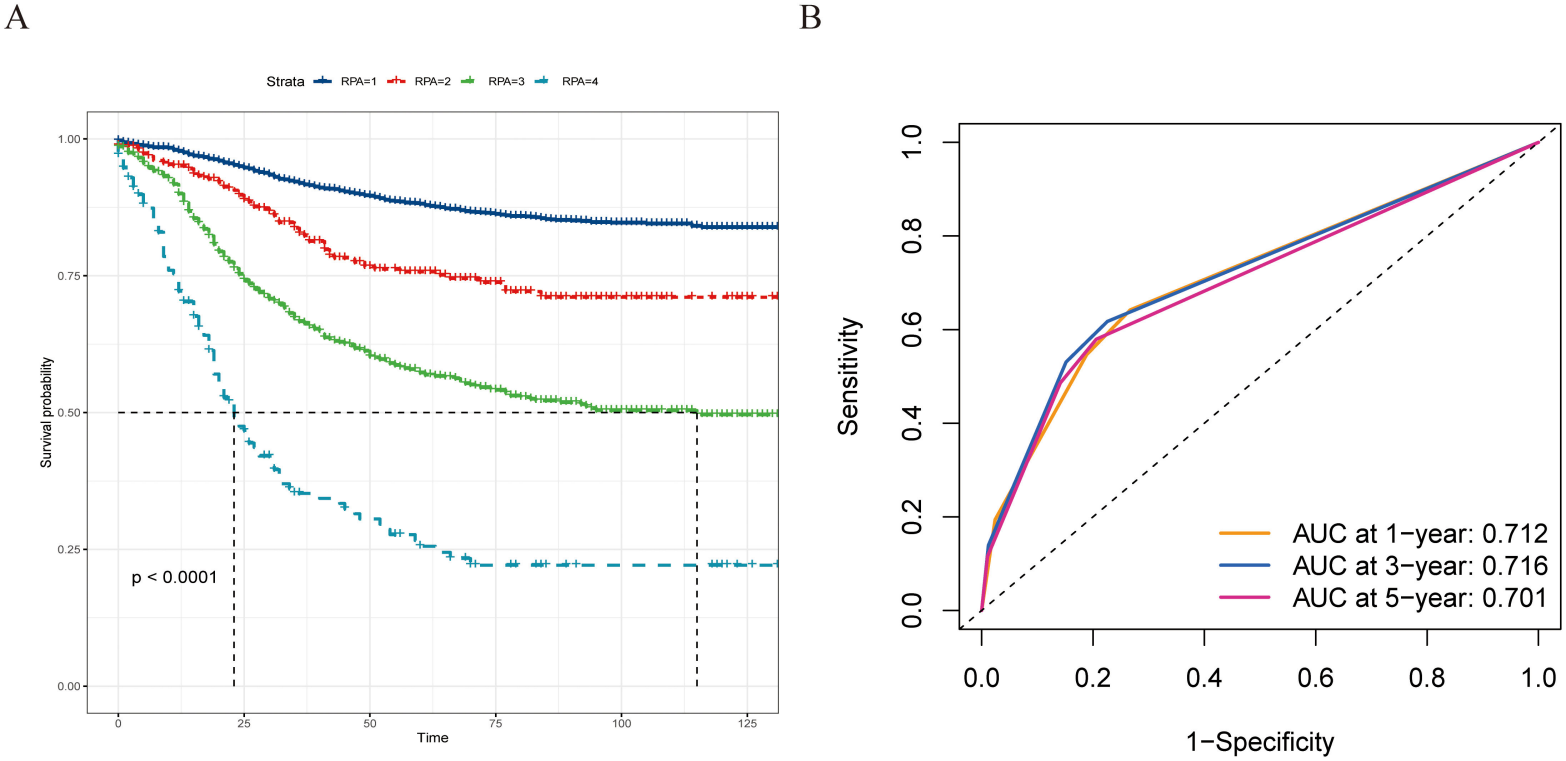
The prognostic evaluation capability of the recursive partitioning analysis staging system, and survival differences were observed across staging subgroups. **(A)** Kaplan–Meier curves evaluating CSS according to the novel staging system. **(B)** ROC curve of the 1-, 3-, and 5-year CSS prediction of the novel staging system (AUC in the bottom right of the figure). ROC, receiver operating characteristic curve; CSS, cancer-specific survival; AUC, the area under the ROC curve.

On the basis of our findings, we proposed a novel prognostic staging system using RPA (**Figure 5A**). The RPA staging system consisted of stages I–IV (**Figure 5B**). Kaplan–Meier curves demonstrated survival differences between the groups (**Figure 6A**). Furthermore, we assessed the predictive performance of the RPA staging system using ROC analysis. The AUC values for predicting the 1-, 3-, and 5-year CSS were 0.712, 0.716, and 0.701, respectively (**Figure 6B**).

## Discussion

This study included patients with colon MAC from the population-based SEER database, and the results demonstrated that LONT outperformed pT, pN, and pTNM stages in predicting 1-, 3-, and 5-year CSS and OS. Cox regression analysis identified LONT as an independent prognostic factor for CSS and OS. Furthermore, LONT was stratified into six subgroups, and multiple subgroup analyses revealed that LONT effectively differentiated the patient survival outcomes. Finally, a novel prognostic staging system was developed using the RPA. These findings provide valuable information to clinicians regarding the management of patients with colon MAC.

MAC is a histological subtype of colon cancer (5, 13–15). Patients with colon MAC are typically diagnosed with advanced disease (T3 or T4 stage) and lymph node metastasis (16, 17). The prognosis for colon MAC remains debatable. Several studies have linked MAC to poor outcomes (18–21). However, some studies indicate that, after adjusting for TNM staging, survival outcomes for MAC are comparable to those of adenocarcinoma (22, 23). Therefore, further evaluation of MAC prognosis is of clinical significance.

T staging and lymph node status are important prognostic factors and are integral components of the TNM staging system. Current research reveals that clinical outcomes vary among patients with the same TNM stage. This could be attributed to the limitation of N staging in reflecting the extent of lymph node dissection, as it only counts the PLNs. Standardized lymph node dissection is a surgical requirement for colon cancer. The inclusion of negative lymph nodes in the LONT calculation partially reflects the extent of lymph node dissection and metastasis. This may explain the improved prognostic predictive ability of LONT. Xie et al. (9) introduced the concept of LONT, which stands for log ^(NLNs+1)/T stage^. This metric represents the T-stage-adjusted negative lymph nodes that reflect the extent of lymph node dissection in gastric cancer. In their study, LONT was independently correlated with gastric cancer prognosis. Yang et al. (24) also confirmed that LONT is a robust prognostic predictor in resectable gastric adenocarcinoma. Furthermore, the prognostic value of LONT has been validated in thyroid cancer without metastases (10) and bladder cancer (11). However, the prognostic value of LONT in colon MAC remains unclear. In this study, lower LONT was associated with poorer prognosis, and LONT was identified as an independent risk factor for CSS and OS in patients with colon MAC. Subgroup analysis of LONT revealed significant survival differences in the advanced pT (pT3 and pT4 stages), pN, pTNM II, and pTNM III stages. This indicates that some patients exhibited favorable outcomes in the pTNM II and pTNM III stages. Identification of these patients is clinically significant for patient management. However, LONT was unable to differentiate the prognosis in the pTNM I stage, probably due to the lower risk of early T-stage lymph node metastasis.

Given the superior performance of LONT in prognostic stratification, a new prognostic staging system was developed using RPA. RPA is used in tumor risk stratification to assist clinical decision-making by analyzing clinicopathological characteristics, making it a practical tool for the prognostic staging of various tumors (25–28). In this study, RPA was used to develop a novel RPA staging system (RPA stages I–IV) by combining the pT stage and LONT. RPA staging is performed clinically on the basis of the pathology of patients with colon MAC. Typically, a lower LONT indicates a poorer prognosis in patients with colon MAC. However, we identified some patients with lower LONT and favorable outcomes using RPA. Patients with pT2 and LONT6 (−0.7 < LONT ≤ 0.1) or pT1-T2 and LONT5 exhibited better clinical outcomes, thereby boosting the confidence of these patients to overcome the disease. Moreover, the newly established RPA staging system demonstrated a strong predictive performance for 1-, 3-, and 5-year CSS. This finding is clinically significant, aiding clinicians in devising personalized follow-up plans and providing patients with the assurance and confidence needed to overcome their condition.

Although we have elucidated the prognostic value of LONT in colon MAC for the first time, our study has certain limitations. First, despite being a population-based study, this was a retrospective study. Second, the SEER database lacks potential prognostic factors, including lymphovascular invasion, perineural invasion, and tumor budding. This limits our ability to assess the impact of lymphovascular invasion, perineural invasion, and tumor budding on the prognosis of patients with colon MAC, as well as to perform risk stratification based on these characteristics. Therefore, large-scale prospective studies are required to validate our findings further and clinically apply the RPA staging system to patients with colon MAC.

## Conclusions

LONT serves as a robust prognostic marker for patients with colon MAC. A lower LONT was associated with poorer clinical outcomes. A novel staging system that combines the pT stage and LONT was developed using RPA. The RPA staging system facilitates personalized risk stratification of patients, thereby aiding clinical decision-making.

## Data Availability

The original contributions presented in the study are included in the article/supplementary material. Further inquiries can be directed to the corresponding author.
